# Endoscopic Endonasal Anterior Skull Base Resection for a SMARCB1-Deficient Sinonasal Carcinoma Arising From the Olfactory Cleft: A Case Report

**DOI:** 10.7759/cureus.116459

**Published:** 2026-09-17

**Authors:** Yuichi Teranishi, Yuki Hasebe, Ayako Kinoshita, Tomoaki Nakano

**Affiliations:** 1 Otorhinolaryngology and Pediatric Otorhinolaryngology, Yodogawa Christian Hospital, Osaka, JPN; 2 Otolaryngology and Head and Neck Surgery, Osaka Metropolitan University, Osaka, JPN

**Keywords:** anterior skull base resection, endoscopic endonasal surgery, olfactory cleft, postoperative radiotherapy, skull base reconstruction, smarcb1-deficient sinonasal carcinoma

## Abstract

A switch/sucrose non-fermentable (SWI/SNF)-related matrix-associated actin-dependent regulator of chromatin subfamily B member 1 (SMARCB1)-deficient sinonasal carcinoma is a rare, aggressive malignancy characterized by loss of nuclear expression of the SMARCB1 protein, also known as integrase interactor 1 (INI1). A 66-year-old man presented with recurrent right-sided epistaxis. Computed tomography and magnetic resonance imaging demonstrated a 50 × 15 × 36 mm mass centered in the right olfactory cleft and extending into the ethmoid sinus. The tumor abutted the anterior skull base, with suspected cribriform plate invasion but no overt intracranial extension. Biopsy showed an undifferentiated carcinoma with loss of SMARCB1/INI1 expression, and the tumor was staged as cT3N0M0. Endoscopic endonasal resection was performed, including unilateral anterior skull base and dural resection to obtain an adequate surgical margin. The skull base defect was reconstructed using a dural substitute, fascia lata, and a vascularized nasoseptal flap. Although the tumor was fully mobilized as a single specimen, it fragmented during transnasal extraction, precluding accurate histopathological assessment of the surgical margins. No dural invasion was identified. Postoperative intensity-modulated radiotherapy was delivered to a total dose of 60 Gy. The patient remained free of recurrence eight months after surgery. This case suggests that endoscopic anterior skull base resection combined with postoperative radiotherapy may provide favorable short-term disease control in resectable SMARCB1-deficient sinonasal carcinoma arising from the olfactory cleft. In high-grade malignancies centered near the cribriform plate, anterior skull base resection should be considered to obtain an adequate surgical margin, even in the absence of overt radiographic intracranial extension, although the extent of resection must be individualized.

## Introduction

A switch/sucrose non-fermentable (SWI/SNF)-related matrix-associated actin-dependent regulator of chromatin subfamily B member 1 (SMARCB1)-deficient sinonasal carcinoma is a rare sinonasal malignancy characterized by loss of SMARCB1 protein expression. The SMARCB1 protein, also known as integrase interactor 1 (INI1), is a core component of the SWI/SNF chromatin-remodeling complex. This carcinoma was independently described by Agaimy et al. and Bishop et al. in 2014 [1,2]. In the fifth edition of the World Health Organization Classification of Head and Neck Tumours, it is recognized as a distinct entity within the category of SWI/SNF complex-deficient sinonasal carcinomas [3]. It frequently presents as locally advanced disease and is associated with poor outcomes; however, no standard treatment has been established because of its rarity [4].

Clinical recognition is challenging because the presenting symptoms overlap with those of more common inflammatory and neoplastic sinonasal conditions [4]. Histopathological diagnosis can also be difficult because the tumor may resemble other poorly differentiated or undifferentiated carcinomas, including nonkeratinizing squamous cell carcinoma and sinonasal undifferentiated carcinoma [2,3]. Immunohistochemical assessment of INI1 expression, interpreted together with tumor morphology and epithelial differentiation, is therefore important for accurate classification [2,3].

We report a case of SMARCB1-deficient sinonasal carcinoma centered in the olfactory cleft that was treated with endoscopic endonasal anterior skull base resection, skull base reconstruction, and postoperative radiotherapy. We focus on the diagnostic evaluation and the rationale for selecting anterior skull base resection despite the absence of overt radiographic intracranial extension.

This article was previously presented as a meeting abstract at the 38th Annual Meeting of Japanese Society for Skull Base Surgery on July 9, 2026 at Tokyo, Japan.

## Case presentation

A 66-year-old man presented with recurrent right-sided epistaxis. At 62 years of age, he had received an immune checkpoint inhibitor and chemotherapy for squamous cell carcinoma of the lung, achieving a complete radiographic response. Treatment was discontinued because of immune-related adverse event-associated lung injury, and he had subsequently remained under surveillance without recurrence.

Approximately three months before his initial visit to our department, the patient began experiencing recurrent right-sided epistaxis. He was referred to our department by the Department of Respiratory Medicine. Nasal endoscopy revealed an irregular, reddish, friable tumor in the superior right nasal cavity (Figure 1A).

**Figure 1:**
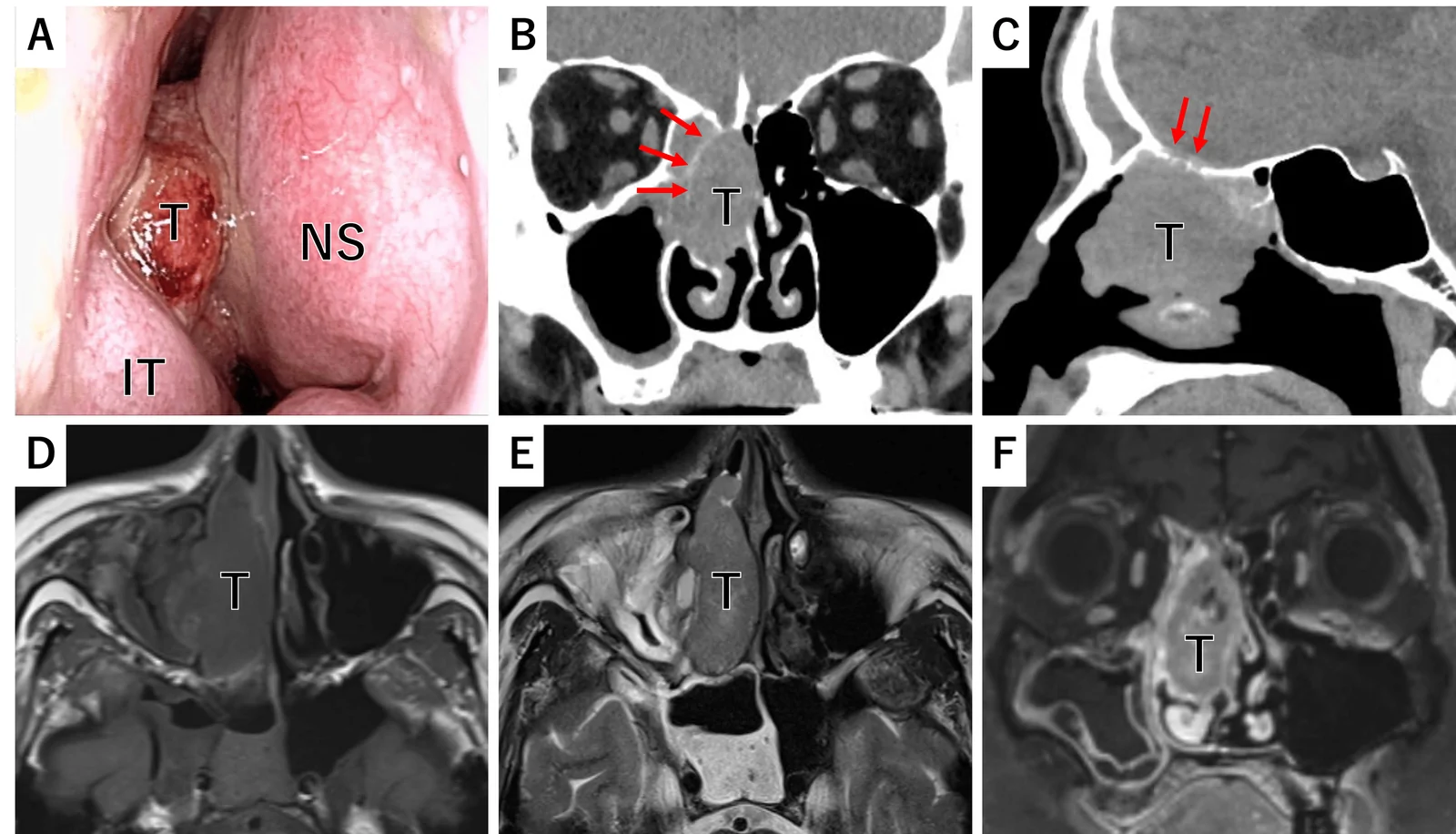
Initial endoscopic and radiological findings (A) Nasal endoscopy revealed an irregular, reddish, friable tumor occupying the superior right nasal cavity. (B) Coronal computed tomography demonstrated a soft-tissue mass extending from the superior right nasal cavity into the ethmoid sinus. The arrow indicates the middle turbinate, which was displaced laterally by the tumor. Focal thinning of the right cribriform plate was also observed. (C) Sagittal computed tomography showed that the tumor extended anteriorly to the inferior margin of the frontal sinus and posteriorly to the anterior wall of the sphenoid sinus. The tumor was closely adjacent to the anterior skull base, and the arrow indicates focal thinning of the cribriform plate. (D-F) Axial T1-weighted, axial T2-weighted, and coronal contrast-enhanced T1-weighted magnetic resonance images, respectively, demonstrated a 50 × 15 × 36 mm mass centered in the right olfactory cleft and extending into the ethmoid sinus. The tumor was hypointense on both T1- and T2-weighted images and showed diffuse contrast enhancement. It abutted the anterior skull base without overt intracranial extension. IT, inferior turbinate; NS, nasal septum; T, tumor.

Computed tomography of the paranasal sinuses demonstrated a soft-tissue mass extending from the superior right nasal cavity into the ethmoid sinus. The middle turbinate was displaced laterally, suggesting that the tumor was centered in the olfactory cleft (Figures 1B-1C). No overt orbital invasion was identified; however, focal thinning of the right cribriform plate raised suspicion of tumor invasion. Magnetic resonance imaging demonstrated a mass measuring approximately 50 × 15 × 36 mm, centered in the right olfactory cleft and extending from the superior nasal cavity into the ethmoid sinus. The tumor abutted the anterior skull base, but no overt intracranial extension was identified (Figures 1D-1F).

Biopsy revealed undifferentiated tumor cells with enlarged round nuclei proliferating in solid nests within a fibrous stroma (Figures 2A, 2B).

**Figure 2:**
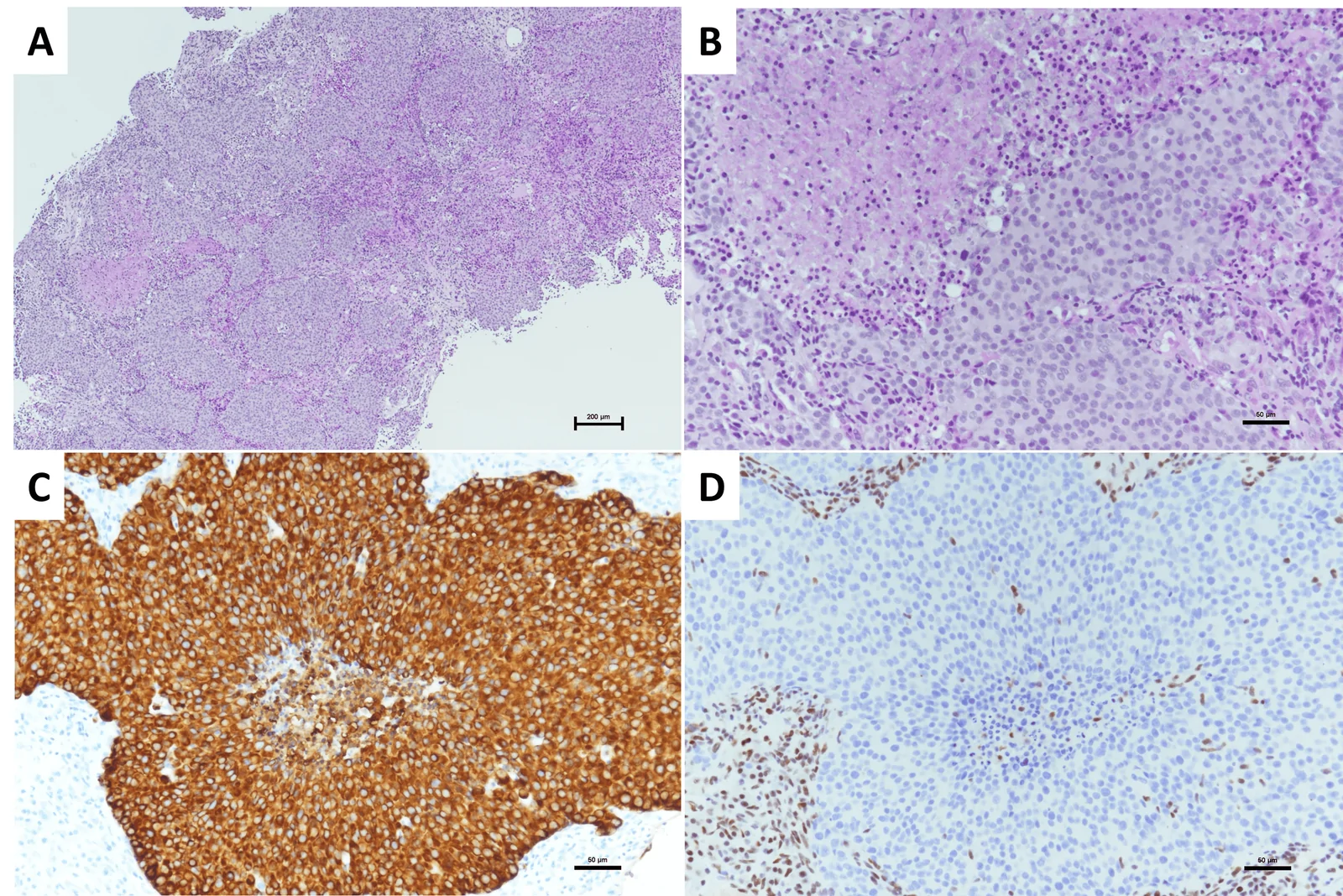
Histopathological and immunohistochemical findings (A) Hematoxylin and eosin staining at low magnification (original magnification, ×50) showed numerous solid tumor nests within a fibrous stroma. (B) At high magnification (original magnification, ×400), the tumor consisted of undifferentiated epithelial cells with enlarged round nuclei. (C) The tumor cells were positive for pancytokeratin. (D) Immunostaining for SMARCB1, also known as integrase interactor 1 (INI1) and BAF47, showed loss of nuclear expression in the tumor cells.

Immunohistochemical staining showed that the tumor cells were positive for pancytokeratin (Figure 2C), cytokeratin (clone CAM5.2), and sex-determining region Y-box transcription factor 10 (SOX10) and negative for thyroid transcription factor 1, p40 (the N-terminally truncated isoform of tumor protein p63), cytokeratin 5/6, insulinoma-associated protein 1, synaptophysin, chromogranin A, S-100 protein, cyclin-dependent kinase inhibitor 2A (p16), and isocitrate dehydrogenase 1. Nuclear expression of Brahma-related gene 1-associated factor 47 (BAF47), also known as INI1, was lost (Figure 2D).

Based on these findings, the tumor was diagnosed as SMARCB1-deficient sinonasal carcinoma. Fluorodeoxyglucose positron emission tomography/computed tomography demonstrated intense uptake in the right sinonasal tumor, with a maximum standardized uptake value of 17.1. No cervical lymph node or distant metastases were identified, and the tumor was clinically staged as cT3N0M0.

Because the tumor was considered resectable, surgery was selected as the initial treatment for local control. The procedure was performed under general anesthesia using an endoscopic endonasal approach. Because the tumor was centered in the olfactory cleft and closely abutted the anterior skull base, unilateral right anterior skull base resection was planned to obtain an adequate surgical margin.

First, septoplasty was performed through a left modified Killian incision. The posterior bony nasal septum was removed, and the sphenoid sinus was reached through a transseptal approach. The bilateral sphenoid sinuses were then opened into a single cavity. A Draf type III frontal sinusotomy was subsequently performed, and bilateral superior lateral anterior pedicle flaps were elevated [5]. The nasolacrimal ducts and lacrimal sacs were exposed bilaterally. The nasal beak and frontal sinus floor were drilled, creating a common cavity between the bilateral frontal sinuses. The bone of the right medial orbital wall was included with the resection specimen, while the periorbita was preserved.

Intraoperative frozen-section analysis showed no tumor involvement of the inferior right septal mucosa. A right nasoseptal flap based on the posterior septal branch of the sphenopalatine artery was therefore harvested. The left septal mucosa was also free of tumor involvement and was preserved.

The right anterior skull base bone and crista galli were removed to expose the dura mater (Figure 3A).

**Figure 3:**
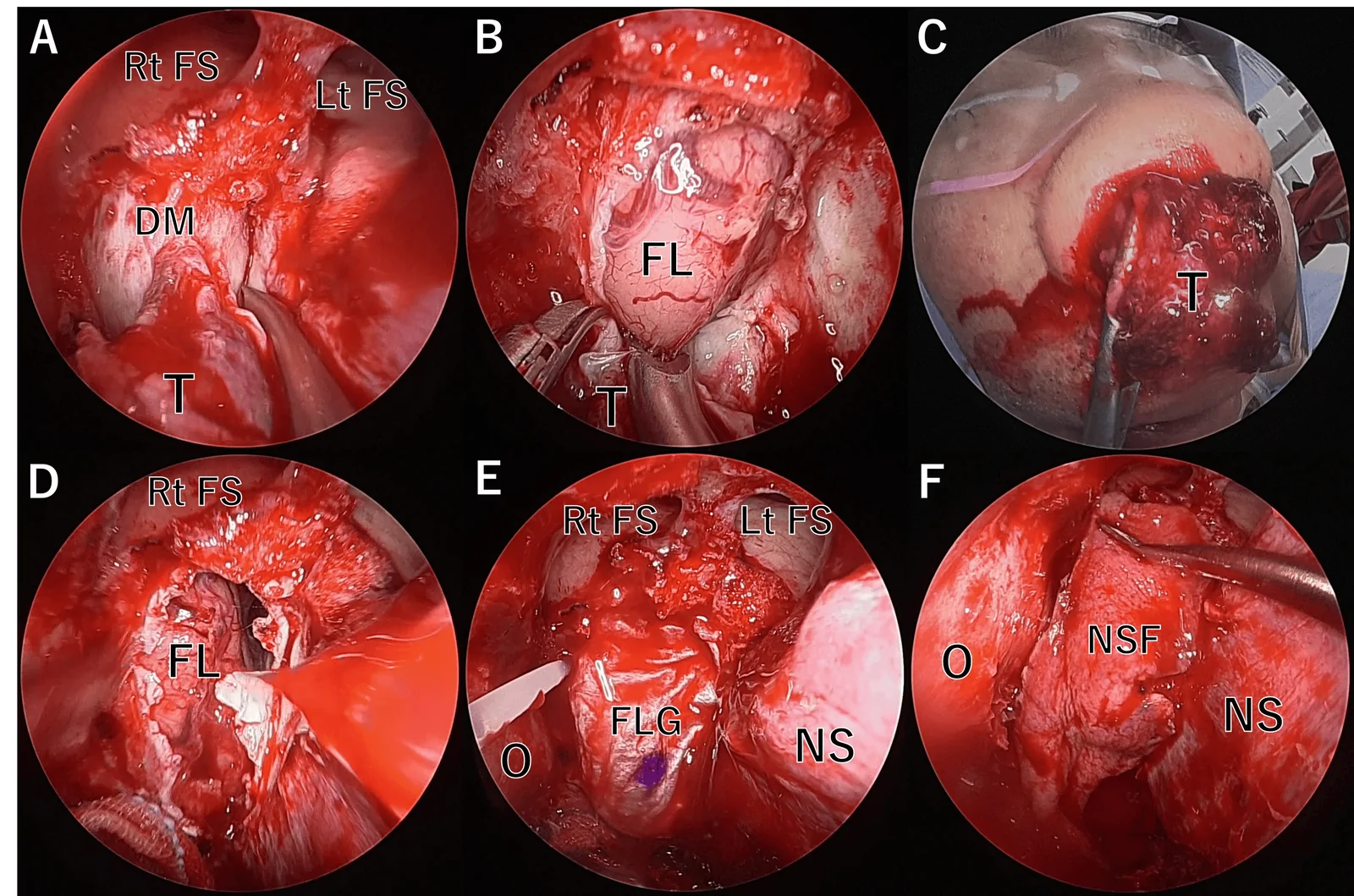
Intraoperative findings and multilayer skull base reconstruction (A) After Draf type III frontal sinusotomy and removal of the right anterior skull base bone and crista galli, the dura mater was exposed. (B) The dura mater was incised, exposing the frontal lobe. (C) The resected tumor specimen was extracted transnasally. (D) Right anterior skull base defect after tumor removal. (E) After placement of an intradural dural substitute, a fascia lata graft was positioned extradurally over the defect. (F) Reconstruction was completed with a right vascularized nasoseptal flap. DM, dura mater; FL, frontal lobe; FLG, fascia lata graft; FS, frontal sinus; NS, nasal septum; NSF, nasoseptal flap; O, orbit; T, tumor.

The lateral edge of the dural resection was placed just medial to the right medial orbital wall, and the dural incision was initiated anteriorly (Figure 3B). Because no tumor attachment was identified at the posterior end of the ethmoid sinus, this level was selected as the posterior transection line. The tumor was circumferentially dissected and fully mobilized as a single specimen. It was then extracted transnasally (Figure 3C), during which the specimen fragmented into several pieces.

The dural defect measured approximately 2 × 1 cm (Figure 3D). Multilayer skull base reconstruction was performed using DuraGen® Dural Graft Matrix (Integra LifeSciences Corporation, Plainsboro, NJ, USA) as an intradural inlay, followed by an extradural fascia lata graft (Figure 3E) and coverage with the right nasoseptal flap (Figure 3F). The bilateral superior lateral anterior pedicle flaps were used to cover the wound created on the anterior-wall side of the drilled frontal sinus floor, including areas of exposed periosteum.

The postoperative course was uneventful, without cerebrospinal fluid leakage or other complications. The histopathological findings of the resection specimen were consistent with those of the biopsy specimen, confirming the final diagnosis of SMARCB1-deficient sinonasal carcinoma. Because the specimen had fragmented during transnasal extraction, accurate histopathological assessment of the surgical margins was not possible. No dural invasion was identified.

Given the high-grade nature of the tumor, its high risk of local recurrence, and the inability to accurately assess the surgical margins, postoperative intensity-modulated radiotherapy was initiated two months after surgery. A total dose of 60 Gy in 30 fractions was delivered to the right sinonasal surgical bed, with the dose reduced to 54 Gy in regions adjacent to the optic nerve. At eight months after surgery, the patient remained free of recurrence and continued to be followed on an outpatient basis.

## Discussion

SMARCB1-deficient sinonasal carcinoma accounted for seven of 140 consecutive sinonasal carcinomas (5.0%) in the series reported by Bishop et al. and seven of 149 consecutive sinonasal carcinomas (4.7%) in the series reported by Li et al., and previous reports have estimated that it accounts for approximately 2%-7% of sinonasal carcinomas [2,3]. However, because of its heterogeneous histological appearance and the likelihood that some tumors were previously diagnosed as other poorly differentiated or undifferentiated carcinomas, the true prevalence may be underestimated.

The pooled analysis by Lee et al. included 120 previously reported cases and eight cases from their institution, for a total of 128 patients. T4a and T4b tumors accounted for 41.4% and 39.1% of cases, respectively, meaning that 80.5% of patients presented with T4 disease. The median overall survival was 39.0 months, and the one-, two-, and three-year overall survival rates were 84.3%, 62.9%, and 51.8%, respectively [4]. In multivariable analysis, T4b disease was the only independent adverse prognostic factor, highlighting the importance of recognizing this disease as early as possible and proceeding promptly to definitive treatment [4].

The SMARCB1 tumor-suppressor gene is located on chromosome 22q11.23, and its protein product, INI1, is a core component of the SWI/SNF chromatin-remodeling complex. Inactivation of SMARCB1 results in loss of nuclear INI1 expression in tumor cells. The histological appearance of SMARCB1-deficient sinonasal carcinoma is variable. In a series of 39 cases reported by Agaimy et al., 23 tumors (59%) exhibited basaloid morphology resembling nonkeratinizing squamous cell carcinoma or undifferentiated carcinoma, whereas 14 tumors (36%) exhibited plasmacytoid or rhabdoid morphology [6]. Pancytokeratin expression was detected in 38 cases (97%), while p63 expression was detected in 20 of the 36 tumors evaluated (55%), demonstrating variable expression [6].

The differential diagnosis includes nonkeratinizing squamous cell carcinoma, sinonasal undifferentiated carcinoma, myoepithelial carcinoma, nonintestinal-type adenocarcinoma, and nuclear protein in testis (NUT) carcinoma [2,3,6]. In poorly differentiated or difficult-to-classify sinonasal malignancies, the morphological findings should guide the selection and interpretation of an immunohistochemical panel that includes INI1 [2,3,6]. Individual markers should not be interpreted in isolation because SMARCB1-deficient sinonasal carcinomas can variably express squamous or neuroendocrine markers [3,6]. In the present case, positivity for pancytokeratin and CAM5.2 supported epithelial differentiation. Negative staining for p40 and cytokeratin 5/6 did not support squamous differentiation, while the absence of insulinoma-associated protein 1, synaptophysin, and chromogranin A expression did not support neuroendocrine differentiation. Loss of nuclear BAF47 (INI1) expression, interpreted together with the undifferentiated morphology and the overall immunophenotype, supported the diagnosis of SMARCB1-deficient sinonasal carcinoma.

No standard treatment has been established for this disease. In the 128-patient analysis by Lee et al., 79 patients underwent curative-intent resection, 72 received postoperative treatment, and 96 received multimodality treatment [4]. In patients with resectable disease, curative-intent resection followed by radiotherapy or chemoradiotherapy has commonly been used. In patients with unresectable or highly advanced disease, treatment options have included induction chemotherapy followed by surgery or chemoradiotherapy, as well as definitive chemoradiotherapy [4].

However, curative-intent resection itself was not evaluated as a prognostic factor in the analysis by Lee et al., and induction treatment, postoperative treatment, and multimodality treatment were not significantly associated with overall survival. Therefore, the superiority of any particular treatment strategy has not been established. Current treatment decisions are individualized with reference to treatment strategies used for other high-grade sinonasal malignancies and the clinical experience accumulated at each institution.

In the present case, the tumor was considered amenable to curative resection at the cT3N0M0 stage, and surgery was selected as the initial treatment. Postoperative treatment was considered because of the high-grade nature of the tumor and the inability to accurately assess the surgical margins after fragmentation of the specimen during extraction. There is limited disease-specific evidence demonstrating an additional benefit from adding chemotherapy to postoperative radiotherapy. Considering the patient's history of immune-related adverse event-associated lung injury, the difficulty of undergoing prolonged hospitalization, and his preference for outpatient treatment, postoperative radiotherapy alone was selected rather than postoperative chemoradiotherapy.

Although no overt intracranial extension was identified radiographically in the present case, the tumor was centered in the olfactory cleft and closely abutted the anterior skull base. The lateral displacement of the middle turbinate on imaging, together with the absence of tumor involvement of the nasal floor and inferior nasal septum at surgery, suggested that the tumor arose from the olfactory cleft.

Ziai et al. evaluated 37 patients with sinonasal malignancies that encroached upon or eroded the bony anterior skull base without overt dural invasion on preoperative imaging. Among 13 patients who underwent dural resection and had evaluable histopathological findings, seven (54%) had occult dural invasion. The negative-margin rate was higher in patients who underwent dural resection than in those who did not (90% vs. 56%, p=0.02) [7]. However, analysis of the entire cohort did not demonstrate a significant improvement in local control or survival following dural resection. The heterogeneity of the tumor types and the small sample size should therefore be considered when interpreting these findings [7].

Geltzeiler et al. conducted a multicenter study of 224 patients with olfactory neuroblastoma. Among 66 patients without radiographic evidence of skull base invasion, eight (12.1%) had occult pathological involvement of the dura or olfactory bulb/tract [8]. Although these findings were obtained from patients with olfactory neuroblastoma and cannot be directly applied to SMARCB1-deficient sinonasal carcinoma, they indicate that malignant tumors arising around the olfactory cleft may exhibit microscopic dural invasion or occult extension to the olfactory bulb or tract even in the absence of overt radiographic intracranial extension.

In the present case, a locally aggressive tumor closely abutted the cribriform plate. We therefore considered that preoperative imaging alone could not completely exclude microscopic dural invasion or microscopic intracranial extension along the olfactory nerve pathway. The right anterior skull base bone and dura were consequently resected to obtain an adequate surgical margin. Although no dural invasion was identified histopathologically, anterior skull base resection was considered a reasonable approach in view of the limitations of imaging and the importance of obtaining negative surgical margins.

Nevertheless, neither the study by Ziai et al. [7] nor that by Geltzeiler et al. [8] specifically investigated SMARCB1-deficient sinonasal carcinoma. Furthermore, the present report describes only a single case. Therefore, these findings cannot be generalized to support dural resection for all olfactory cleft tumors without radiographic intracranial extension. The extent of resection should be determined individually according to tumor histology, extent of disease, and the surgical margin that can be obtained.

The patient remained free of recurrence eight months after surgery. However, the follow-up period remains short, and both local recurrence and distant metastasis have been reported after treatment of this disease. Careful long-term surveillance is therefore required. Because SMARCB1-deficient sinonasal carcinoma is rare and most available evidence consists of case reports or small retrospective series, further accumulation of cases is necessary to clarify the optimal indications for surgery, extent of resection, role of postoperative treatment, and role of chemotherapy.

## Conclusions

SMARCB1-deficient sinonasal carcinoma is a rare sinonasal malignancy that frequently presents as locally advanced disease and for which no standard treatment has been established. In the present case, although no overt intracranial extension was identified radiographically, the tumor was centered in the olfactory cleft and closely abutted the anterior skull base. Endoscopic endonasal anterior skull base resection and multilayer skull base reconstruction were performed to obtain an adequate surgical margin. The short-term outcome following surgery and postoperative radiotherapy was favorable. However, careful long-term surveillance remains necessary. Further multicenter studies with long-term follow-up are needed to clarify the optimal extent of surgical resection and the role of adjuvant therapy in SMARCB1-deficient sinonasal carcinoma.
